# Rapid development of a COVID‐19 care planning decision‐aid for family carers of people living with dementia

**DOI:** 10.1111/hex.13552

**Published:** 2022-06-18

**Authors:** Emily West, Pushpa Nair, Narin Aker, Elizabeth L. Sampson, Kirsten Moore, Jill Manthorpe, Greta Rait, Kate Walters, Nuriye Kupeli, Nathan Davies

**Affiliations:** ^1^ Marie Curie Palliative Care Research Department University College London London UK; ^2^ Department of Primary Care and Population Health, Centre for Ageing Population Studies, Royal Free Campus University College London London UK; ^3^ Department of Psychological Medicine, Royal London Hospital East London NHS Foundation Trust London UK; ^4^ National Ageing Research Institute Parkville Victoria Australia; ^5^ NIHR Policy Research Unit in Health and Social Care Workforce King's College London, Strand London UK; ^6^ NIHR Applied Research Collaborative (ARC) South London King's College London, Strand London UK

**Keywords:** coproduction, COVID‐19, decision making, dementia, engagement

## Abstract

**Introduction:**

COVID‐19 has disproportionately affected people living with dementia and their carers. Its effects on health and social care systems necessitated a rapid‐response approach to care planning and decision‐making in this population, with reflexivity and responsiveness to changing individual and system needs at its core. Considering this, a decision‐aid to help families of persons with dementia was developed.

**Objectives:**

To coproduce with people living with dementia, and the people who care for them, a decision‐aid for family carers of people living with dementia, to support decisions during the COVID‐19 pandemic and beyond.

**Methods:**

Semi‐structured interviews were undertaken in 2020 with: (1) staff from two English national end‐of‐life and supportive care organizations; and (2) people living with dementia and family carers. Simultaneously, a rapid review of current evidence on making decisions with older people at the end of life was undertaken. Evidence from these inputs was combined to shape the decision‐aid through a series of workshops with key stakeholders, including our patient and public involvement group, which consisted of a person living with dementia and family carers; a group of clinical and academic experts and a group of policy and charity leads.

**Results:**

The rapid review of existing evidence highlighted the need to consider both process and outcome elements of decision‐making and their effects on people living with dementia and their families. The qualitative interviews discussed a wide range of topics, including trust, agency and confusion in making decisions in the context of COVID‐19. The decision‐aid primarily focussed on care moves, legal matters, carer wellbeing and help‐seeking.

**Conclusions:**

Combining different sources and forms of evidence was a robust and systematic process that proved efficient and valuable in creating a novel decision‐aid for family carers within the context of COVID‐19. The output from this process is an evidence‐based practical decision‐aid coproduced with people living with dementia, family carers, clinical and academic experts and leading national dementia and palliative care organizations.

**Patient or Public Contribution:**

We worked with people living with dementia and family carers and other key stakeholders throughout this study, from study development and design to inclusion in stakeholder workshops and dissemination.

## 1 INTRODUCTION

COVID‐19 has disproportionately affected people living with dementia, with over 26% of deaths from COVID‐19 occurring in this population.^1^ This group is especially vulnerable to COVID‐19, and at particular risk of more severe illness, complications and death.^2^ In particular, people from ethnic minority groups were particularly affected by COVID‐19^3^ and are also underrepresented in dementia research.^4^ Navigating existing health systems within this context has been fraught. Ever‐changing demands on health systems and knock‐on effects of changing public policies have necessitated a rapid‐response approach to care planning and decision‐making.^5^

The key to decision‐making in these circumstances is reflexivity and responsiveness to changing individual and system needs. At various points throughout the pandemic in the United Kingdom, visiting has been limited in both general hospitals and care homes, telemedicine has been prioritized over in‐person appointments, routine appointments have been cancelled and communication between hospitals and families has drastically changed. Within these difficult circumstances, complex care decisions still have to be made. If a person becomes unwell, family members may have to make rapid decisions concerning hospital admission, social distancing, do not attempt cardiopulmonary resuscitation orders, ceilings of care and treatment cessation.^6^ In England and Wales, the Mental Capacity Act 2005 highlights the importance of support in specific decision‐making in persons with diminished mental capacity.^7^ For people lacking capacity, including some people living with dementia, the role of family members in supporting the decision‐making process is key—and for some, specific powers will have been authorized (such as a Lasting Power of Attorney (LPA) covering health and welfare decision‐making) or an Advance Decision^8^ will be in place. Otherwise, the ultimate responsibility for decisions legally resides with the treating clinicians.

In England and Wales, the government enables advanced or proxy decision‐making by adults with the capacity to put these in place, including those living with dementia. For those who then lose the capacity in making decisions, family carers or other nominated individuals can act as proxy decision‐makers if appointed under the Mental Capacity Act 2005.^7^ This can be a demanding if rewarding role under normal circumstances, but the COVID‐19 context meant that many usual means of accessing reliable evidence‐based support and guidance to make difficult care decisions were lessened due to redeployment of healthcare professionals and lack of face‐to‐face access to general practitioners, social workers and day centre staff. Such decision‐making affects not only the immediate care and wellbeing of the person living with dementia but also the possible ongoing grief, or simple physical and emotional wellbeing of the carer.^6^ Decision‐making tools, such as decision‐aids, are an effective means of support for persons with dementia and their carers,^9^ though currently there are more single‐issue tools (e.g., in relation to deciding the optimal ‘ceilings’ or limits of care) than overarching multitopic tools.^10^

At the beginning of the pandemic, family carers took on more care responsibilities;^11^ however, decision‐making support for this group was in short supply. Considering this, we aimed to rapidly and rigorously coproduce a decision‐aid to help families of people living with dementia that was informed by a combination of qualitative data and evidence synthesis. In particular, we anticipated that the decision aid would need to focus on decisions, such as place of care, place of death and hospitalization; however, it was important to explore these with people living with dementia, family carers and professionals. This paper discusses the rapid development process of this decision‐aid and reflects on the process, content and development.

## 2 METHODS

### 2.1 Design

We used a coproduction approach with key stakeholders including our family carers' public and patient involvement (PPI) group to rapidly develop a decision‐aid. Coproduction has been used throughout healthcare and research to develop resources for not just practitioners but also families and patients themselves. Coproduction uses a partnership approach in which the research team, practitioners and end users (i.e., patients and carers) have equal participation in decision‐making and the development is thus driven by the end users, which in this case were family carers.^12^, ^13^ Coproduction ensures that the voices of those who are experts in the area, both through experience and professionally, together with those who will be using the decision‐aid are incorporated into the development. Coproduction is recommended as an approach for the development of decision‐aids.^14^ We used a variety of asynchronous and synchronous methods, including workshops, individual meetings and written feedback to coproduce our decision aid.^15^ This ensured that the voices of all end users were heard. This information was combined with data from a literature review and individual semi‐structured interviews with people living with dementia, family carers and professionals to produce our decision aid.

We have reported the contents and the development process of the decision‐aid using the International Patient Decision Aid Standards (IPDASi v 4.0)^16^ (see Table A1).

### 2.2 Underpinning theory

We used two theoretical frameworks and models to guide the development of the decision‐aid: (1) decision‐making model from our rapid review and (2) Ottawa Decision Support Framework.

#### 2.2.1 Decision‐making theory

Our theoretical model from the rapid review formed the basis of the approach we took to develop the decision‐aid. Our model highlights different needs and aspects of decision‐making, from considering informational, access and cultural needs to facilitating conversations and access to formal options, such as advance care planning and LPA. It also reinforces the iterative and constantly negotiated nature of the decision‐making process. This is particularly important within the context of COVID‐19 as it balances rapidly changing informational structures, contexts and options with a consistent process and approach to decision‐making and care planning. Steps within this process include ongoing access to information, considering values and preferences, facilitating and enabling conversations, empowering diversity, navigating options, managing carer wellbeing, legal aspects of decision‐making and being responsive to change.

#### 2.2.2 Ottawa Decision Support Framework

We also used the Ottawa Decision Support Framework (ODSF)^17^ to shape the content of the decision‐aid and prioritize best practices in aiding decisions that are constantly changing. This Framework recommends that underpinning any decisional support, including decision‐aids, there should be processes to clarify decisions and decisional needs, provide facts, clarify personal values, guide deliberation and communication and monitor/facilitate progress. Alongside these, we used O'Cathain's taxonomy of intervention development to guide coproduction.^12^ This includes a synthesis of 18 actions in conception, planning, designing, creating, refining, documenting and planning for future evaluation of interventions, across a taxonomy of different approaches (see the aims of the workshops section below for how O'Cathain's work informed our procedure).

### 2.3 Procedure

We used four evidence sources: (1) rapid review and synthesis of current evidence,^18^ (2) qualitative interviews with charity helpline staff,^19^ (3) qualitative interviews with people living with dementia and their carers from ethnic minority groups^20^, ^21^ and (4) synthesized evidence from charities and national organizations reporting on COVID‐19 and dementia/older people.

These evidence sources were summarized and synthesized in a matrix (see Table 1 e.g., matrix) to highlight key themes and patterns, including areas where there are gaps in support and information at the time. We combined this knowledge with previous work conducted with members of the research group as a foundation for the decision‐aid.^22^

**Table 1:** Example of matrix used to collate and synthesize evidence

**Decision or challenge**	**Example decision/challenge**	**Factors influencing decision**	**Rapid review**	**Professional interviews**	**Carer and PWD interviews**	**Other COVID guidance**	**Decision‐aid section**
Example: Place of care and place of death	Should care workers come into the person's home.	Fear from person with dementia, fear from family carers, influence of media, struggling at home, risk and feelings of guilt.	Being prepared and having a sense of control are important for making decisions.	Uncertainty about where is the best place to be and should they live with their parent, or move them into their home during this crisis.	Many adhered to self‐imposed lockdowns and felt socially isolated.	Alzheimer's Disease International.^23^	Thinking about any existing Advance Care Plans.
	Should the person move to a care home?						Managing care at home.
					Family was fearful of transmitting the virus and visiting even if outside.		
							Supporting someone in a care home.
			Decisions are iterative and change over time as illness and capacities change.				
					Fears of care workers transmitting the virus when visiting, which led to stopping services.		
							Should they go to the hospital if they are unwell?
				People are worried that care workers will bring the virus into their homes, so people are ‘locking down’ and trying to do everything themselves.			
			Ensure sensitivity to individuals' culture and background.				
				Do not want to call the health service helpline (111) in case an ambulance comes and admits them to the hospital.			
				Fear of asking for help because of what they have seen on news and what may happen if the person leaves home.			
				Letting care workers in or support is about risk management.			

#### 2.3.1 Summary of findings from earlier phases of the overall project

##### 2.3.1.1 Rapid review

A rapid review of current evidence on making decisions with older people at the end of life was undertaken.^18^ This review was performed using the 2017 WHO guidance for rapid reviews for the production of actionable evidence, due to the rapidly changing nature and fast response needed to the early pandemic.^24^ We chose to perform a review‐of‐reviews, including meta‐analyses, to maximize available evidence, identifying 10 reviews covering both qualitative and quantitative original studies (full methods are available in the published review).^18^ Papers focussed on older people—those over the age of 65, professionals, carers and the general population who were concerned with caring for people over the age of 65 years. We focussed on decisions surrounding care and place of care/place of death in this population.

These themes were synthesized and further analysed iteratively by the research team, and a decision‐making model was developed based on these findings. This model highlights different needs and aspects of decision‐making, from informational, access and cultural needs to facilitating conversations and ensuring access to legal options, such as advance care planning and LPA.

There was a split in focus between papers that focussed on the process of decision‐making and those that concentrated on outcomes of decision‐making. In papers covering the process of making decisions, themes included: factors affecting decision‐making, emotional aspects of decision‐making, capacity in decision‐making and ethnic minority group experiences in making decisions. When looking at outcomes of decision‐making, the main themes included: place of death, decisions over time and what affects decisions made. Full findings of the review are reported elsewhere.^18^

Both the themes identified and the decision‐making model informed the topic guide for the qualitative interviews. The findings from the rapid review and qualitative interviews were used to develop the decision‐aid.

##### 2.3.1.2 Qualitative interviews

The foundation of the decision‐aid was a qualitative study using semi‐structured interviews with staff from national end‐of‐life and supportive care organizations, and interviews with carers and people living with dementia from ethnic minority backgrounds, analysed using thematic analysis.

Interviews were undertaken between May–June 2020, and lasted 30–60 min. Staff were recruited through convenience sampling from two UK charities that work in dementia and end‐of‐life care. Various sources were used to recruit people living with dementia and their carers from ethnic minority groups, including relevant local/national carer organizations, online dementia research recruitment websites (e.g., Join Dementia Research) and social media (e.g., Twitter).

The findings from the qualitative interviews, as well as being written up as a research paper,^19^, ^20^, ^21^ formed the foundational data for the development of the decision‐aid; guiding topics covered, resources identified and areas of need to address.

There was an overarching theme of fear and anxiety in the interviews with carers and people living with dementia from ethnic minority groups. Participants discussed difficulties with shopping for food, which caused considerable anxiety, particularly linked to the risk of exposing the person with dementia to COVID‐19 through contact with others in shops. Both family carers and people living with dementia reported feeling socially isolated. Family carers in particular felt strained without their usual outlets for support or interaction, which meant their identity was impacted by not being able to be anything other than a carer. A feeling of being part of a community was affected by a lack of engagement with places of worship, or the ability to attend religious and cultural celebrations for many. Participants discussed difficulties with adapting to COVID‐19, particularly for those who lacked awareness and found not being able to go out or the changing rules frustrating. Finally, for many a major source of stress was the need to make decisions about care during a period where less support was available. The full findings are published elsewhere.^20^

Interviews with professionals identified concerns and areas of decision‐making that family carers were calling charity helplines about, including: concerns about care moves; uncertainty in engaging with support and seeking help; pandemic‐motivated care planning; maintaining the wellbeing of the person living with dementia and issues around trust, loss of agency and confusion.^19^

#### 2.3.2 Stakeholder workshops

Together with all stakeholders, including our PPI group (person living with dementia and family carers), healthcare professionals and policy makers, we synthesized the information from the rapid review and qualitative studies to produce a decision‐aid for family carers and people with dementia to use when making difficult decisions in the context of COVID‐19.

##### 2.3.2.1 Aims of workshops

Using O'Cathain's taxonomy of intervention development,^12^ the aims of workshops were: to understand stakeholders' experiences and perspectives surrounding decision‐making in this context; to understand areas of most need that the decision‐aid should address to change behaviour around decision‐making; to understand the role of the decision‐aid within real‐life decisions and contexts; to ensure appropriate resources to enhance and promote change surrounding decisions; to generate solutions and identify avenues to success based on the experience of stakeholders; to optimize the content, format and dissemination of the decision‐aid for maximal impact and finally, to refine and prototype the decision‐aid.

##### 2.3.2.2 Composition and schedule of workshops

Two homogenous groups were convened for coproduction, to enable groups of stakeholders to contribute freely without reinforcement of existing power hierarchies or structures perceived—such as the differentiation between professionals, family carers and persons living with dementia. The first group consisted of family carers and a person living with dementia. The second group consisted of professionals. Two workshops were organized, as well as individual sessions. Individual sessions were predominantly used to engage with frontline staff whose competing time demands meant they could not attend group sessions. It can be challenging to involve people living with dementia in coproduction workshops and ensure their voice is heard over the voices of others. Other work has reported people with dementia find these methods difficult to engage with and challenges of being involved in coproduction of interventions.^25^, ^26^ This may be particularly problematic while being conducted remotely online. The person living with dementia in our coproduction workshop was supported by their family carer. For this study, we interviewed people living with dementia separately to ensure their voices were heard.^20^, ^21^ We placed substantial emphasis on this interview data when devising the decision‐aid prototype. Facilitators highlighted in the workshops and in the interview data the key messages from people living with dementia, thus ensuring the voices of people living with dementia were represented in the workshop discussions.

Workshops were held online via Microsoft Teams, a business teleconferencing software, led by a facilitator and cofacilitator. Detailed notes were taken by the cofacilitator throughout the sessions. Each group met one time for 90 min, and stakeholders indicated whether they would be prepared to give written feedback on future drafts via email. We embraced the flexibility of the coproduction methods and approaches, which allowed us to complete this study during a global pandemic, using what has been termed low contact approaches in coproduction.^15^ We used a combination of synchronous and asynchronous approaches, including one‐to‐one meetings and written feedback. This was due to necessity—the need to develop the decision‐aid to meet the rapidly developing COVID‐19 situation, and physical restrictions imposed by the government.

##### 2.3.2.3 Integration of workshops

Based on the initial evidence synthesis, decisions and mitigating factors were mapped out. We used a matrix that not only allowed transparency (see Table 1) but also highlighted the gaps in knowledge and evidence. The research team then iteratively refined these. We produced a draft decision‐aid based on this iterative analysis of data, combined with the structure of the decision‐making model. The draft was informed by a decision‐aid from a linked study,^22^ which was developed for the same population before the COVID‐19 pandemic. This draft decision‐aid, as well as data from the evidence synthesis, was then presented in stakeholder workshops. We encouraged stakeholders to reflect on different elements of decisions pertinent to the situation, and how to approach these. Throughout the workshops, members were asked whether the decision‐aid contained the information that they expected, and members were encouraged to make suggestions for topics not yet covered, or that needed more depth.

Discussions from these workshops were further synthesized and summarized into our evidence matrix and guided further drafts of the decision‐aid. Stakeholders who had agreed to contribute further were contacted by email for feedback.

### 2.4 Ethical approval

Ethical approval is not required for involvement activities, but we sought and received ethical approval for the semi‐structured interviews, which informed the decision‐aid development (University College London Research Ethics Committee 18215/001 and 17623/002).

## 3 DECISION‐AID CONTENT AND STRUCTURE

The decision‐aid is available in paper format or can be accessed and downloaded online here: (https://www.ucl.ac.uk/psychiatry/sites/psychiatry/files/endemic_decision_aid_26_08_20_v.2.pdf). We have split the results section into the various sections of the decision‐aid and described the contents for each section, with reference to the stakeholder workshops and how sections link to the theory and evidence from earlier phases.

### 3.1 Introduction to decision‐aid

The decision‐aid opens with a contact organization section, detailing the names and relationships of those on the form, and relevant professional contacts—left blank for carers to complete as they need. The introduction section then provides a brief overview of the purpose of the decision‐aid, including what decisions may need to be made, clarifying the decision needs as outlined in the ODSF steps.^17^ A contents page allows the decision‐aid to be navigated by sections of interest. This was highlighted as a need in workshops with carers and people living with dementia, who commented that otherwise the document may seem unwieldy if presented as a stepwise task. The inclusion of a contents page allows carers to identify areas of most need for themselves, and engage in the process in a manner most suitable to their need.

### 3.2 COVID‐19 in dementia

The decision‐aid moves on to explain its utility in making decisions, with particular attention to the context of COVID‐19—including symptoms that may help identify COVID‐19 in older people. This was something particularly highlighted during coproduction. Stakeholders in workshops also highlighted the importance of reinforcing existing wishes and care plans because when carers are making decisions in an emergency context it may be difficult to recall these. The decision‐aid also encourages carers to consider existing LPA for the person living with dementia if not already done so. This aligns with the ODSF step of clarifying the personal values of the individual with dementia.

### 3.3 Reflecting on care plans and wishes

**Figure 1:**
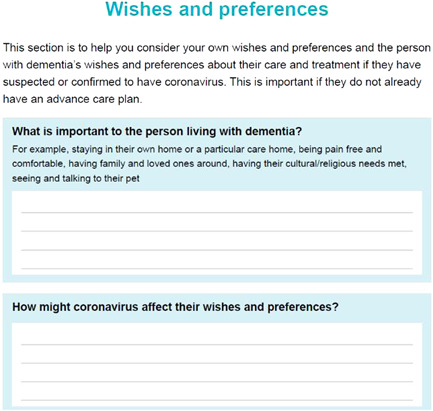
Reflective exercise to prompt carers to consider individual's wishes, values and preferences.

The decision‐aid encourages users to consider practical aspects of care management and delivery. This section contains written information and a reflective exercise to prompt carers into defining what they and the person living with dementia are comfortable with, in terms of interventions, guiding the deliberation and communication (see Figure 1). This was highlighted in the workshop with professionals as being an effective way of helping carers refer back to decisions made if an emergency arises. This section then goes on to detail ways in which help may be delivered, and potential avenues for seeking support, aligning with the ODSF step of providing facts.

### 3.4 Managing care at home

Across the qualitative interviews, participants discussed the uncertainty of engaging with services at home and what support was available to them, with many perceiving there were limited services and support. Professional stakeholders in workshops were keen to stress that support was available at home and that carers should not feel hesitant about asking for support.

### 3.5 Supporting someone in a care home

**Figure 2:**
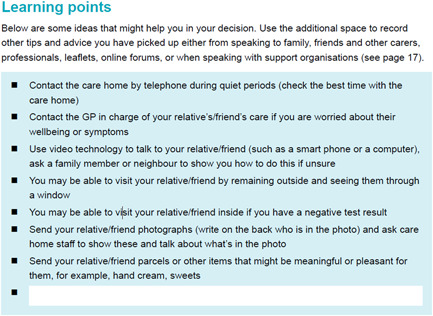
Learning points section for supporting someone in a care home.

This section provided information about how family carers or friends could keep in touch and feel included in the support of someone living in a care home when visiting was not allowed or limited. Several interview participants expressed anxiety about leaving home and visiting relatives in a care home, even when keeping outside and seeing relatives through the window as many were doing. The coproduction groups felt this was very relevant and important to address by providing some learning points about how carers could be supported, including when to contact the care home, and sending parcels to relatives of meaningful or pleasant items, such as photographs and sweets (see Figure 2).

### 3.6 Deciding on hospital admissions

This subject was prominent across workshops. During the time in which this decision‐aid was made, there were widespread concerns about admission to the hospital for those who were particularly clinically vulnerable to COVID‐19, so information and decisions concerning this move were highlighted specifically. Qualitative interviews also illustrated how stressful such admissions were for both people contacting charity helplines, and carers and persons living with dementia from ethnic minority groups who had concerns about not being able to see the person living with dementia once in hospital. These findings were reinforced in the coproduction activities by the PPI group, and by expert stakeholders from health and social care.

### 3.7 Supporting the carer

The remainder of the decision‐aid focuses on support for the carer. This includes a mapping exercise that encourages the carer to consider their existing support network and highlights areas where they may need further input or support. Workshops had highlighted the risk of isolation in carers, so prompting a section that encouraged carers to consider this—and resources to help ameliorate this—to be included. We encouraged carers to contact health and social care professionals to promote their feeling of being supported and engaged and provided activities they could do to foster their wellbeing. The decision‐aid contained sections for carers to write down key topics and questions to discuss with professionals. The decision‐aid concludes with a list of resources available across the United Kingdom that may provide help and support for those caring for people living with dementia in the context of COVID‐19, such as national charities and helplines.

### 3.8 Format and design considerations

Carers from the workshop stressed the importance of needing to make the decision‐aid engaging and accessible to a wide range of carers. It was highlighted that many carers already feel overwhelmed by competing for information, and that fatigue around reading dense documents was widespread. Consequently, we prioritized presenting information in varied ways throughout the document. We used text boxes to break up and highlight particularly pertinent information within large portions of text. Similarly, we used bullet points to emphasize information that did not especially need to all be taken in at once (see Figure 2), and a clear contents page to help carers access the parts most pertinent to them easily.

Several topics throughout the document were presented as writing exercises that required the input of the carer (see Figure 1). This format was chosen to engage carers and facilitate the identification of resources and support specific to individuals. Similarly, some topics were presented as tick‐box exercises, for easy future reference. The exercise that encouraged carers to consider their support networks was formatted as a map‐type diagram to provide a visual means of engaging with this potentially difficult subject. Information was designed to be accessible and written for the average reading age in the United Kingdom (9 years) and designed accessibly.^27^ The ODSF and the model from the rapid review shaped the presentation of information.

## 4 DISCUSSION

This paper presents the rapid development of a decision‐aid for family carers of people living with dementia within the context of COVID‐19. This is the only decision‐aid developed for this population during COVID‐19, and we have reported on the adaptation of methods to ensure rapid development of an applied and practical decision‐aid for implementation, which is still evidence‐based and systematic. This paper has provided an overview of the development of the decision‐aid, including evidence identified through a literature review, qualitative interviews with those providing support from national charity helplines, qualitative interviews with people living with dementia and family carers from ethnic minority groups, and a synthesis of these elements, alongside design undertaken with people with lived experience of living with, or caring for those living with dementia. The decision‐aid covered care planning—particularly around hospital admissions, carer support systems, access to information and contingency considerations.

### 4.1 Decision‐making in dementia

The eventual loss of or fluctuations in decision‐making capacity among people living with dementia and the unpredictable progression of dementia are just two factors that make decision‐making in dementia care complex^28^, ^29^ Research has shown that preparedness and a sense of control are important in making care decisions.^28^, ^30^, ^31^ This is particularly significant within the context of COVID‐19, where navigating healthcare and support systems has been uncertain and unfamiliar. Carers have reported feeling isolated^32^, ^33^ during the pandemic, and many have found it difficult to access support. Our decision‐aid provides a means of engaging with care decisions and supportive resources outside clinical settings, which may not be accessible during different stages of a pandemic. The decision‐aid encourages forward planning in a rapidly changing situation where decisions may have to be made quickly if a person living with dementia's needs change. This promotes preparedness for people living with dementia and carers.

### 4.2 Developing policy in emerging health situations

This decision‐aid was developed remotely in the first few months of the COVID‐19 pandemic, under the first UK national lockdown. This unique set of circumstances brought with it certain challenges. As well as human‐level risk, healthcare services were dealing with rapidly changing needs for service access and allocation, rapid response triaging and increased service utilization. These complex needs were being navigated in an environment where government guidelines and mandates were sometimes unclear and changing frequently. Thus, the process of developing the decision‐aid needed to be done in such a way that overarching messages and approaches would stay stable in the face of changing circumstances, while individual‐specific pieces of guidance (i.e., on hospital admissions) would be able to be reflexive if policies changed. Balancing competing needs of reflexivity and clear messaging has been identified as a key challenge in rapid‐response care planning, where trust is key to successful provision.

Ryan et al.^34^ discuss this balance within the framework of ‘technologies of trust’, whereby ‘openness (a willingness and genuine effort to incorporate multiple perspectives), reflexivity (flexibly responsive to context and the ongoing dialogue) and accountability (taking responsibility for local contexts and consequences)’ are seen as key to developing practical techniques and successful interventions in the context of emergency or rapid response. By including perspectives from people living with dementia, family carers and professionals from practice and academia in the formulation of the decision‐aid, we incorporated openness. Reflexivity was modelled in having multiple and ongoing opportunities for input from all groups involved and accountability by openly responding to and incorporating guidance and comment from stakeholder sources.

### 4.3 Designing the decision‐aid

The decision‐aid was developed through coproduction, an approach to research and production that prioritizes the needs and input of stakeholders to ensure that interventions are relevant to end users.^35^, ^36^ A key strength of the decision‐aid is the involvement of multiple and varied groups of stakeholders during the development process, including people living with dementia, carers, health and social care professionals, and people leading national policy on dementia care. The final format of the decision‐aid provides not only written information but opportunities for carers to engage actively in decisions and wider subjects such as self‐care. The variety of approaches and topics covered in the decision‐aid is a direct result of the diversity of voices heard in coproduction, and is another strength of the decision‐aid. Due to restrictions imposed by the UK lockdown and social restrictions at this point, coproduction was performed digitally via videoconferencing software as well as using a combination of synchronous and asynchronous methods.^15^ This proved a feasible and beneficial method for reaching diverse groups of collaborators but its limitations are acknowledged.

### 4.4 Implications for research

Combining different sources and forms of evidence was efficient and valuable in creating a novel decision‐aid. A major strength of coproduction was the involvement of people living with dementia. Further research looking at the involvement of persons living with dementia in coproduction of tools and interventions should be prioritized. To embody the principles of openness by facilitating the input of many and varied stakeholder groups, it is essential that groups historically considered ‘hard‐to‐reach’ are involved in coproduction of tools that will affect them upon implementation.

The use of rapid methods needs to balance the need for timely interventions with the need to be scientifically robust and evidence grounded. This study offers a road map to achieve this. We encourage others to use the matrix approach to develop interventions as it enables synthesis across a variety of sources and identifies gaps. Our methods can be used in time and resource‐pressured environments. However, we accept that coproduction needs to remain meaningful and beyond pandemic times it will naturally take longer to engage people meaningfully.

### 4.5 Strengths and limitations

This paper provides a comprehensive overview of the rapid development—over the span of 4 months—of a practicable healthcare decision‐aid within the context of a pandemic. The methods undertaken were rapid, but still systematic, rigorous and evidence‐based. We prioritized the inclusion of families and carers from diverse backgrounds, a range of professionals and achieved ‘buy in’ from key organizations. The decision‐aid incorporates a range of information—rapid review, qualitative data, grey literature and evidence synthesis. The matrix approach used is transparent, which is key in rapid development, and also allows for a team approach to the development of the decision‐aid.

Coproduction is a process where it is of the utmost importance to engage groups well. This is something that is difficult to perform rapidly. Outside the context of the pandemic, coproduction engagement would be difficult to enact on these timelines. However, as much of our coproduction work was completed online this may have excluded the voices of those who were not able to communicate using online platforms. This process would have been strengthened with greater input from people living with dementia in coproduction workshops, including a separate workshop or individual meetings with people living with dementia. However, we were able to interview people living with dementia, which informed the discussions and the evidence presented in codesign workshops and thus the resulting decision‐aid.

The decision‐aid is available online to download and print to use in paper format. However, this may limit the use of the decision‐aid for those who are not able to access or use the internet. We have shared the decision‐aid with leading carer and dementia organizations who are able to support people to receive a copy of the decision‐aid if they cannot access it online themselves.

### 4.6 Implications for research, policy and practice

Upon publication, the decision‐aid was adopted by NHS England and other leading healthcare organizations.^37^ The decision‐aid has been widely disseminated as an aid to help navigate the healthcare system under the limitations of the pandemic. Adapted or similar‐in‐format decision aids have the potential to help other groups of carers, or other people making complex healthcare decisions in different contexts. The widespread national adoption of the decision‐aid suggests the potential for utilizing such tools more widely.

The decision‐aid has the potential to be used for general care planning, decision‐making in acute situations as a catalyst to engage in conversations about death and dying and in decisions around discharge planning. The document has been adopted by some hospitals as part of their discharge documents, to encourage carers to think ahead about future care and potential options.

There are also potential benefits for carers and people living with dementia. Good decision‐making has been shown to reduce decisional conflict,^38^, ^39^ which, in turn, can have a positive effect on the quality of life, care and death as well as wider risks of stress and anxiety.

Due to the time‐limited nature of this study, it was not possible to pilot test the decision‐aid or evaluate implementation. Future research could focus on assessing outcomes and developing strategies for promoting uptake.

## 5 CONCLUSIONS

The output of this process was an evidence‐based decision‐aid that was produced rapidly with leading experts in relevant fields in the United Kingdom. Upon publication, it was adopted by national governing bodies and cosigned by leading charities and health and social care organizations, as well as individual clinical and care settings. Thus, rapidly developing a decision‐making tool and utilizing and synthesizing evidence from a variety of sources are feasible and actionable approaches to tool development, particularly within a health emergency context.

## AUTHOR CONTRIBUTIONS

Nathan Davies, Nuriye Kupeli, Kirsten Moore and Elizabeth L. Sampson conceived the idea and design of the study. Nathan Davies, Nuriye Kupeli, Kirsten Moore, Elizabeth L. Sampson, Pushpa Nair, Emily West, Jill Manthorpe, Greta Rait and Kate Walters acquired funding. Emily West, Pushpa Nair and Narin Aker acquired data and led the analysis. All authors contributed to the interpretation of the results, drafted and finalized the manuscript and approved the version to be published and agreed to be accountable for all aspects of the work.

## CONFLICT OF INTEREST

The authors declare no conflict of interest.

## Data Availability

Data sharing is not applicable to this article as no datasets were generated or analysed during the current study.

## APPENDIX A

See Table A1.

**Table A1:** IPDASi v 4.0 checklist

Item	Included (when applicable)
*Qualifying criteria*	
Describes health condition or problem.	X
Explicitly states the decision that needs to be considered.	X
Describes the options available.	X
Describes the positive features (benefits/advantages) of each option.	X
Describes the negative features (harms, side effects, or disadvantages) of each option.	X
Describes what it is like to experience the consequences of the options (physical, psychological, social).	X
*Certification criteria*	
Shows the negative and positive features of options in equal detail.	X
Provides citations to the evidence selected.	(How it was developed are detailed including who was involved)
Provides a publication date.	X
Provides an update policy.	
Provides information about the levels of uncertainty around event or outcome probabilities.	
Provides information about the funding source used for development.	X
Describes what the test is designed to measure.	N/A
Describes the next steps typically taken if the test detects the condition.	N/A
Describes the next steps if the condition is not detected.	N/A
Has information about the consequences of detecting the condition that would never have occurred if screening had not been done (lead‐time bias).	N/A
*Quality criteria*	
The patient decision aid describes the natural course of the health condition or problem, if no action is taken.	X
The patient decision aid makes it possible to compare the positive and negative features of the available options.	X
The patient decision aid provides information about outcome probabilities associated with the options (i.e., the likely consequences of decisions).	X
The patient decision aid specifies the defined group (reference class) of patients for whom the outcome probabilities apply.	
The patient decision aid specifies the event rates for the outcome probabilities.	
The patient decision aid allows the user to compare outcome probabilities across options using the same time period (when feasible).	
The patient decision aid allows the user to compare outcome probabilities across options using the same denominator (when feasible).	
The patient decision aid provides more than one way of viewing the probabilities (e.g., words, numbers, and diagrams).	X
The patient decision aid asks patients to think about which positive and negative features of the options matter most to them (implicitly or explicitly).	X
The patient decision aid provides a step‐by‐step way to make a decision.	X
The patient decision aid includes tools like worksheets or lists of questions to use when discussing options with a practitioner.	X
The development process included a needs assessment with clients or patients.	X
The development process included a needs assessment with health professionals.	X
The development process included review by clients/patients not involved in producing the decision support intervention.	X
The development process included review by professionals not involved in producing the decision support intervention.	X
The patient decision aid was field‐tested with patients who were facing the decision.	
The patient decision aid was field‐tested with practitioners who counsel patients who face the decision.	X
The patient decision aid (or associated documentation) describes how research evidence was selected or synthesized.	X
The patient decision aid (or associated documentation) describes the quality of the research evidence used.	
The patient decision aid includes authors'/developers' credentials or qualifications.	X
The patient decision aid (or associated documentation) reports readability levels.	
There is evidence that the patient decision aid improves the match between the preferences of the informed patient and the option that is chosen.	N/A
There is evidence that the patient decision aid helps patients improve their knowledge about options' features.	N/A
The patient decision aid includes information about the chances of having a true‐positive test result.	N/A
The patient decision aid includes information about the chances of having a true‐negative test result.	N/A
The patient decision aid includes information about the chances of having a false‐positive test result.	N/A
The patient decision aid includes information about the chances of having a false‐negative test result.	N/A
The patient decision aid describes the chances the disease is detected with and without the use of the test.	N/A
